# Supplementary material for: MTaxi: A comparative tool for taxon identification of ultra low coverage ancient genomes
Source: Open Res Eur. 2023 Sep 29;2:100. Originally published 2022 Aug 22. [Version 3] doi: 10.12688/openreseurope.14936.3 (PMC10565424; doi:10.12688/openreseurope.14936.3)
Supplement: Peer review history

# Reviewer report: Reviewer response for version 3

**DOI:** 10.21956/openreseurope.18011.r35391

**Published:** 2023-10-10

**Authors:** Burak Yelmen

**Affiliations:** 1 Estonian Biocentre, Institute of Genomics, University of Tartu, Tartu, Tartu County, Estonia; 2 Laboratoire Interdisciplinaire des Sciences du Numérique, Paris-Saclay University, Gif-sur-Yvette, Île-de-France, France

I thank the authors for their clarifications, I believe they improved the manuscript. Although the performance metrics used were defined differently (such as false negatives, which should correspond to misidentified cases for the negative class in binary classification), since the terminology is now clearly defined in the methods, I'm in favour of indexing the article.

Is the rationale for developing the new software tool clearly explained?

Yes

Is the description of the software tool technically sound?

Partly

Are sufficient details of the code, methods and analysis (if applicable) provided to allow replication of the software development and its use by others?

Partly

Is sufficient information provided to allow interpretation of the expected output datasets and any results generated using the tool?

Partly

Reviewer Expertise:

My main research fields are population genetics, genomics, deep learning and generative modelling. My previous work involved demographic analysis of human populations based on modern and ancient genomic data. In addition, one of my ongoing research projects involve ancient DNA simulations.

I confirm that I have read this submission and believe that I have an appropriate level of expertise to confirm that it is of an acceptable scientific standard.

# Reviewer report: Reviewer response for version 2

**DOI:** 10.21956/openreseurope.17517.r33586

**Published:** 2023-08-30

**Authors:** Burak Yelmen

**Affiliations:** 1 Estonian Biocentre, Institute of Genomics, University of Tartu, Tartu, Tartu County, Estonia; 2 Laboratoire Interdisciplinaire des Sciences du Numérique, Paris-Saclay University, Gif-sur-Yvette, Île-de-France, France

Revised manuscript is substantially improved with additional analyses and authors clarified almost all of the questions raised previously. However, there are two minor points I believe still need clarification for the performance metric calculation.

 Authors' accuracy metric defined in the methods sections seems to actually correspond to F1 score (harmonic mean of recall and precision). In the manuscript, authors use the term "accuracy", which could be confusing to the readers since these two metrics are different and F1 score doesn't take into account true negatives (TN) so one class is omitted. I believe accuracy might be a more suitable metric in this case because correct predictions from both classes are taken into account.

 Secondly, authors define recall as #(correctly identified cases)/#(unidentified + identified cases) and precision as (#correctly identified cases)/(#identified cases). Usually, recall is calculated as TP/(TP + FN) and precision as TP/(TP + FP). I'm not sure if I understand what corresponds to what here. I believe the confusion probably stems from the fact that these metrics are normally used for binary classification with a positive and a negative class and in authors' case, there is also the unidentified class. However, I think it's important to clarify this confusion because "recall" in authors' definition becomes "accuracy" if #(correctly identified cases) corresponds to TP + TN (in the case of sheep/goat, correctly identified sheep + correctly identified goat) and the denominator (unidentified + identified cases) seems to cover all cases. Or if #(correctly identified cases) corresponds to TP only, then recall would not be meaningful (since denominator seems to cover everything). Or maybe there is some issue with the denominator? Similarly, there is an ambiguity for the precision too. These would also affect the calculation of F1 score.

Is the rationale for developing the new software tool clearly explained?

Yes

Is the description of the software tool technically sound?

Partly

Are sufficient details of the code, methods and analysis (if applicable) provided to allow replication of the software development and its use by others?

Partly

Is sufficient information provided to allow interpretation of the expected output datasets and any results generated using the tool?

Partly

Reviewer Expertise:

My main research fields are population genetics, genomics, deep learning and generative modelling. My previous work involved demographic analysis of human populations based on modern and ancient genomic data. In addition, one of my ongoing research projects involve ancient DNA simulations.

I confirm that I have read this submission and believe that I have an appropriate level of expertise to confirm that it is of an acceptable scientific standard, however I have significant reservations, as outlined above.

# Reviewer report: Reviewer response for version 2

**DOI:** 10.21956/openreseurope.17517.r33585

**Published:** 2023-07-18

**Authors:** Kevin G. Daly

**Affiliations:** 1 Smurfit Institute of Genetics, Trinity College Dublin, Dublin, Ireland

The changes made by the authors have improved the manuscript and accessibility of the MTaxi tool.

Is the rationale for developing the new software tool clearly explained?

Yes

Is the description of the software tool technically sound?

Partly

Are sufficient details of the code, methods and analysis (if applicable) provided to allow replication of the software development and its use by others?

Partly

Is sufficient information provided to allow interpretation of the expected output datasets and any results generated using the tool?

Partly

Reviewer Expertise:

I work in the palaeogenomics of domestic animals, particularly goat. I have experience working with samples of extremely low endogenous DNA and sequencing reads, and have worked on species assignment of such samples. I was also responsible for the goat genomic data included in analyses in the text.

I confirm that I have read this submission and believe that I have an appropriate level of expertise to confirm that it is of an acceptable scientific standard.

# Reviewer report: Reviewer response for version 1

**DOI:** 10.21956/openreseurope.16140.r31295

**Published:** 2023-05-23

**Authors:** Burak Yelmen

**Affiliations:** 1 Estonian Biocentre, Institute of Genomics, University of Tartu, Tartu, Tartu County, Estonia; 2 Laboratoire Interdisciplinaire des Sciences du Numérique, Paris-Saclay University, Gif-sur-Yvette, Île-de-France, France

In this study, authors propose a method for taxonomic assignment of a sample to either of two closely related species using ancient mitochondrial DNA (mtDNA). The method focuses on mtDNA positions with transversion substitutions between the two candidate species, assigns mtDNA reads from the sample in question to either candidate based on the read composition and uses a binomial test on these assignments to identify the true taxon. Authors test their approach both with simulated and real ancient mtDNA with varying coverages for sheep/goat and donkey/horse candidate pairs and compare their results with comparative alignment.

 The manuscript is well written and covers the related literature background with appropriate references. It characterizes the problem related to the taxonomic identification of ancient samples and provides a useful solution for specific cases. I think the study is sound overall and can benefit the aDNA field, especially since the proposed method does not require high quality reference genomes and utilises more abundant mtDNA. However, some minor/moderate revisions could improve the manuscript. Below I provide my comments/questions and propose possible revisions, mainly for clarification purposes. The comments which start with an asterisk (Methods 4, Methods 6, and Results 1) are the points I believe are the most important and especially need to be addressed.

**Abstract**

1. " *...to classify closely related species that are compelling to distinguish...*" -> that are difficult to distinguish?

**Introduction**

1. Some citations could be helpful for the paragraph where alignment issues are discussed [1,2].
2. " *...and ambiguity which can significantly...*" -> typographical error

**Methods**

1. Figure 1: Different colors for the nucleotides in the first and second panel can be confusing to the reader. Authors might consider captioning the colors or changing the visual representation of transversion/transition substitutions in the first panel.
2. " *We call these “target sites” and are obtained from pairwise alignment of mitochondrial reference genomes.*" -> grammatical error
3. It sounds reasonable to restrict the analysis to transversions to reduce the effects of DNA damage and possible homoplasy but this causes a significant reduction in statistical power. For future studies, authors may consider including more substitutions to see how the identification is affected. One idea might be to use models for ancient DNA damage patterns (such as [3], based on non-independent C-T substitutions) to select the target positions for analysis.
*Related to the previous comment, PMDtools with --deamination parameter was used in data processing but it is not clear for what purposes. Was filtering performed for likely deamination with this step?
5. Why different algorithms, BWA mem and BWA aln, were used for ancient and modern genomes? Is it based on literature [4]?
*It could be useful to elaborate on the one-tailed and two-tailed binomial tests used in the study and how unidentified cases were defined based on this in the methods section.
7. For ancient mitogenome simulations, why did authors opt not to simulate with lower coverage (<0.5x)? It could have been interesting to see how the performance changes with lower coverage, since authors also state in the discussion that lower coverage mtDNA is quite common.
8. Could authors elaborate on the rationale to downsample the equid mtDNA to 0.3x to 4x coverages?

**Results**

*Precision and recall measurements might be confusing since there are also unidentified cases. Authors specify no misidentified cases (no false positives) but later in the results report 98% and 99% precision (referring to figure 3c and 3d). What are the false positives in this case? I think there needs to be clear definitions for all the performance metrics used.
2. " *...even at mtDNA coverages ≥0.5x*" -> Did authors mean =0.5x? Or "even" could be omitted. 
3. " *...here the recall was >50% at 0.5x...*" Authors could give the exact percentage for recall here. Relatedly, please also refer to my first comment for the results.
4. Table 2. Caption ("Total assigned reads") does not match the table ("Total reads").
5. " *...would be only 0.0003%...*" I assume this is 0.5^18? I would prefer writing either ~0.00038% or ~0.0004%, not to confuse the readers.
6. " *...except one sheep, which had a coverage lower than 0.3x...*" -> In Table S2, this sheep sample seems to have ~0.1x coverage, why did authors emphasize <0.3?
7. In the comparative alignment section, authors wrote " *...differ only marginally (by ~0.7%) in their sheep vs. goat mismatch proportions.*" and concluded that " *...our results imply that its success may not be guaranteed in all circumstances.*" I believe this conclusion might not be appropriate without statistical backing. 

**Discussion **

1. I think the degree of divergence is an interesting aspect, it could be a future direction to investigate how well the method performs with various degrees of relatedness between reference species. 
2. Related to the previous comment, authors could emphasize the limitations of their method more thoroughly in the discussion. For example, how could the identification be affected if the target sample belongs to a third species? Could it be misidentified or fall under the unidentified category? 

Is the rationale for developing the new software tool clearly explained?

Yes

Is the description of the software tool technically sound?

Partly

Are sufficient details of the code, methods and analysis (if applicable) provided to allow replication of the software development and its use by others?

Partly

Is sufficient information provided to allow interpretation of the expected output datasets and any results generated using the tool?

Partly

Reviewer Expertise:

My main research fields are population genetics, genomics, deep learning and generative modelling. My previous work involved demographic analysis of human populations based on modern and ancient genomic data. In addition, one of my ongoing research projects involve ancient DNA simulations.

I confirm that I have read this submission and believe that I have an appropriate level of expertise to confirm that it is of an acceptable scientific standard, however I have significant reservations, as outlined above.

## References

[1] : Quantifying and reducing spurious alignments for the analysis of ultra-short ancient DNA sequences. *BMC Biol* .2018;16(1) : 10.1186/s12915-018-0581-9 121 10.1186/s12915-018-0581-9 30359256PMC6202837

[2] : Assessing DNA Sequence Alignment Methods for Characterizing Ancient Genomes and Methylomes. *Frontiers in Ecology and Evolution* .2020;8: 10.3389/fevo.2020.00105 10.3389/fevo.2020.00105

[3] : AuthentiCT: a model of ancient DNA damage to estimate the proportion of present-day DNA contamination. *Genome Biol* .2020;21(1) : 10.1186/s13059-020-02123-y 246 10.1186/s13059-020-02123-y 32933569PMC7490890

[4] : Additional evaluations show that specific BWA-aln settings still outperform BWA-mem for ancient DNA data alignment. *Ecol Evol* .2021;11(24) : 10.1002/ece3.8297 18743-18748 10.1002/ece3.8297 35003706PMC8717315

# Response

**Published:** 2023-06-18

**Authors:** Gözde Atağ

**Affiliations:** Middle East Technical University, Turkey

Abstract   "...to classify closely related species that are compelling to distinguish..." -> that are difficult to distinguish?  

**We thank Dr. Yelmen for this suggestion, we now use “difficult” instead of “compelling”.**  

 Introduction   Some citations could be helpful for the paragraph where alignment issues are discussed [1,2].

**We thank Dr. Yelmen for this suggestion, we added the suggested citations where the alignment issues are discussed.**    

 "...and ambiguity which can significantly..." -> typographical error  

**We corrected the sentence as “...an ambiguity which can significantly...".**      

 Methods   Figure 1: Different colors for the nucleotides in the first and second panel can be confusing to the reader. Authors might consider captioning the colors or changing the visual representation of transversion/transition substitutions in the first panel.  

**We captioned the colors to avoid confusion.**

 "We call these “target sites” and are obtained from pairwise alignment of mitochondrial reference genomes." -> grammatical error  

**We corrected the sentence as follows : These “target sites” are obtained from pairwise alignment of mitochondrial reference genomes.**

 It sounds reasonable to restrict the analysis to transversions to reduce the effects of DNA damage and possible homoplasy but this causes a significant reduction in statistical power. For future studies, authors may consider including more substitutions to see how the identification is affected. One idea might be to use models for ancient DNA damage patterns (such as [3], based on non-independent C-T substitutions) to select the target positions for analysis.  

**We agree with Dr. Yelmen about the reduction in statistical power, we discuss this and possible alternative approaches in the Discussion part. The manuscript now reads as follows :** **Although the target sites are restricted to transversion substitutions as a means to prevent the effects of post-mortem transitions and possible homoplasies, this restriction also results in a considerable reduction in statistical power. Alternatively, MTaxi users could choose to use both transition and transversion substitutions by (a) by using aDNA libraries prepared with Uracil-DNA Glycosylase (UDG) treatment to remove the uracils from ancient molecules (1), (b) by masking those positions susceptible to post-mortem damage at read ends (https://github.com/etkayapar/bamRefine), or (c) by accounting for ancient DNA damage patterns using probabilistic models (2, 3). ** 

 *Related to the previous comment, PMDtools with --deamination parameter was used in data processing but it is not clear for what purposes. Was filtering performed for likely deamination with this step?  

**Thank you for pointing out this omission. PMDtools was only used on non-UDG-treated libraries for the purpose of checking the authenticity of the ancient sequences. We now clarify this point under Methods section:** **“In order to confirm the authenticity of the ancient DNA sequences from non-UDG-treated libraries (TEP03, TEP62, TEP83, ULU26, ULU31), we evaluated damage patterns characteristic of aDNA using PMDtools with the “--deamination” parameter; all libraries had >20% C->T and G->A damage at 5’ and 3’ ends, respectively.”**

 Why different algorithms, BWA mem and BWA aln, were used for ancient and modern genomes? Is it based on literature [4]?  

**We thank Dr. Yelmen for pointing this out, and we now explain our rationale in the Ancient DNA Data Processing section, based on literature. The manuscript now reads as follows :** **BWA mem is designed as a highly sensitive aligner for reads >70 bp (4). It has been also shown that BWA aln outperforms BWA mem in ancient DNA sequence alignment in terms of precision and proportion of the reads mapped (5). We therefore used BWA aln for the alignment of ancient DNA sequences and mem for modern DNA sequences. **  

 *It could be useful to elaborate on the one-tailed and two-tailed binomial tests used in the study and how unidentified cases were defined based on this in the methods section.

**We thank Dr. Yelmen for this suggestion. We now elaborate the use of one-tailed and two-tailed binomial tests in the Methods section as follows :** **Here and throughout the study, for the evaluation of the power of MTaxi using samples with known species identity, we use one-tailed binomial tests since we have an a priori hypothesis about each sample’s taxon. Meanwhile, in the method itself we use a two-tailed binomial test assuming both target species are equally likely.** 

 For ancient mitogenome simulations, why did authors opt not to simulate with lower coverage (<0.5x)? It could have been interesting to see how the performance changes with lower coverage, since authors also state in the discussion that lower coverage mtDNA is quite common.

**We thank Dr. Yelmen for this suggestion. We now added results from simulations of ancient mitogenomes at 0.3x and 0.1x, and tested the performance; the results are presented in the updated Figure 3 (see Figure R1 below), and in Results and Discussion. Results showed 100% precision at both coverages, >90% and >50% recall for 0.3x and 0.1x respectively. We also updated the Discussion to suggest that MTaxi can be used with sufficient accuracy at 0.3x coverage.** **Figure R1:** **Binomial test p-values for comparing the proportions of reads assigned to (a) sheep versus goat, and (b) and horse versus donkey.**

 Could authors elaborate on the rationale to downsample the equid mtDNA to 0.3x to 4x coverages?

**Thank you for this point. In fact in Table 3, we only downsampled seven present-day equid genomes, while the three ancient genomes were used as is. When downsampling we aimed at obtaining c.4x, two c.2x, two c.1x, and two 0.5x genomes. We now explain this in the table legend and in Methods.**

 Results   *Precision and recall measurements might be confusing since there are also unidentified cases. Authors specify no misidentified cases (no false positives) but later in the results report 98% and 99% precision (referring to figure 3c and 3d). What are the false positives in this case? I think there needs to be clear definitions for all the performance metrics used.  

**We agree with Dr. Yelmen about this ambiguity. We now define our metrics under Methods , in the newly added “Performance metric calculation” section as follows :** **We measured the performance of MTaxi using three metrics; precision, recall and accuracy. Precision was calculated as (#correctly identified cases)/(#identified cases), recall was calculated as #(correctly identified cases)/#(unidentified +identified cases). Accuracy was estimated using the F-scores, calculated as (2 × Precision × Recall)/(Precision + Recall)). **    

**We also corrected the results related to the current Figure S1c (previous Figure 3c), which indeed had precision 100%. We thank the reviewer for noticing this error.**  

 "...even at mtDNA coverages ≥0.5x" -> Did authors mean =0.5x? Or "even" could be omitted.

**We omitted “even” from the sentence**.

   "...here the recall was >50% at 0.5x..." Authors could give the exact percentage for recall here. Relatedly, please also refer to my first comment for the results.  

**We now report the exact percentages as “..here the recall was 52% at 0.5x, but reached 97% at 1x coverage.”**

 Table 2. Caption ("Total assigned reads") does not match the table ("Total reads").  

**We thank Dr. Yelmen for this suggestion. We corrected the table caption as "Total assigned reads".**

 "...would be only 0.0003%..." I assume this is 0.5^18? I would prefer writing either ~0.00038% or ~0.0004%, not to confuse the readers.  

**We now provide the percentage as ~0.00038%.**

 "...except one sheep, which had a coverage lower than 0.3x..." -> In Table S2, this sheep sample seems to have ~0.1x coverage, why did authors emphasize <0.3?  

**We thank Dr. Yelmen for pointing this out. The link in the manuscript is not updated to the latest version of the supplementary tables, in which the coverage is reported as 0.22x. We now updated the link to the latest version in Zenodo (https://doi.org/10.5281/zenodo.8051744).**

 In the comparative alignment section, authors wrote "...differ only marginally (by ~0.7%) in their sheep vs. goat mismatch proportions." and concluded that "...our results imply that its success may not be guaranteed in all circumstances." I believe this conclusion might not be appropriate without statistical backing.

**Evaluating the power of comparative mapping would require a study on its own. Here we just wanted to point out that this approach may have limitations. However, we see Dr. Yelmen’s point. We therefore changed the sentence as "...our results raise the possibility that its success may not be guaranteed in all circumstances."**

 Discussion   I think the degree of divergence is an interesting aspect, it could be a future direction to investigate how well the method performs with various degrees of relatedness between reference species.

**Thank you for this suggestion. We added a sentence that reads as:** **“** **It may be especially interesting to investigate how MTaxi performs depending on the divergence time between sister taxa.”**

 Related to the previous comment, authors could emphasize the limitations of their method more thoroughly in the discussion. For example, how could the identification be affected if the target sample belongs to a third species? Could it be misidentified or fall under the unidentified category?

**Thank you for this point. Following Dr. Daly’s suggestion, we aligned n=3 ancient horse samples to sheep and goat reference genomes and ran MTaxi. None of the horse samples showed a significant affinity to either sheep or goat (p>0.05). The results are presented in the newly added section titled “Performance under alternative scenarios” (Table R1, Table S5).** **Table R1.** **MT** **axi results on horse genome data of known species identity (default approach), where horse is not among the candidate species.**

 Horse samples were aligned to sheep and goat references, and the analysis was performed with transversion substitutions between sheep and goat.

**Sample ID** **Taxon** **mtDNA** **coverage** **Total** **assigned** **reads** **Goat** **reads** **Sheep** **reads** **p value** **Identified** **taxon**

 Cdy2 Horse 0.319088 22 14 8 **>0.05** **-** Vhr031 Horse 0.72599 4 2 2 **>0.05** **-** Vhr102 Horse 1.29874 29 17 12 **>0.05** **-**            

**References**  

1. Rohland N, Harney E, Mallick S, Nordenfelt S, Reich D. Partial uracil–DNA–glycosylase treatment for screening of ancient DNA. Philosophical Transactions of the Royal Society B: Biological Sciences. 2015;370(1660):20130624. doi:10.1098/rstb.2013.0624  
2. Link V, Kousathanas A, Veeramah K, Sell C, Scheu A, Wegmann D. Atlas: Analysis tools for low-depth and ancient samples [Internet]. Cold Spring Harbor Laboratory; 2017 [cited 2023 Jun 8]. Available from: https://www.biorxiv.org/content/10.1101/105346v1
3. Skoglund P, Malmström H, Omrak A, Raghavan M, Valdiosera C, Günther T, et al. Genomic diversity and admixture differs for stone-age Scandinavian foragers and Farmers. Science. 2014;344(6185):747–50.
4. Li H. Aligning sequence reads, clone sequences and assembly contigs with BWA-Mem [Internet]. 2013 [cited 2023 Jun 8]. Available from: https://doi.org/10.48550/arXiv.1303.3997
5. Oliva A, Tobler R, Llamas B, et al.: Additional evaluations show that specific BWA-aln settings still outperform BWA-mem for ancient DNA data alignment. Ecol Evol. 2021; 11 (24): 18743-18748

# Reviewer report: Reviewer response for version 1

**DOI:** 10.21956/openreseurope.16140.r29949

**Published:** 2022-08-25

**Authors:** Kevin G. Daly

**Affiliations:** 1 Smurfit Institute of Genetics, Trinity College Dublin, Dublin, Ireland

Atağ and colleagues report on MTaxi, a bioinformatic tool that compares the alignments of a single sample to two different mitochondrial sequences. A likely species identity is assigned among the pair based on a binomial test of reads overlapping transversion variant sites between the two sequences. Analysis can also be restricted to reads which map to both reference sequences, although improvement of false positive rates appears negligible in the sequence pairs tested. The tool is developed to aid species identification when working with archaeological material which may be one of two difficult-to-distinguish species, and when endogenous DNA preservation is poor.

 The manuscript is exceptionally well written in its description of the broader field and the specific research question to be addressed. Citations are appropriate and support the arguments made in the text. The tool reported has use in solving specific problems in the genetic analysis of archaeozoological material when resources are limited, which is growing increasingly important as the field democratizes and broadens. The method has an excellent false positive rate and the incorrect species is unlikely to be assigned even for low coverage samples, assuming one of the two species is the correct one. There are also obvious ways that this approach could be extended in the future e.g. testing a set of reference mitochondria rather than just a pair. This could be very useful in large screening studies e.g. analyses of sediment samples.

 As the manuscript stands, I would recommend several changes that can be made to the text to improve clarity to the readers regarding the method. The authors might consider a revision of their manuscript to take on some or all of these suggestions, but the method itself appears robust. A major point of clarity is the input files required; while bam and fastqs are self explanatory, the files defining the transversion variant sites are less so. Should each file (for species 1 and 2) have the same number of variant sites, with line *n* in file 1 matching the same variant site in line *n* of file2? This could be clarified in the Github repository ( https://github.com/goztag/MTaxi ).

 One additional request is for the inclusion in the Github repository of example data e.g. a small subset of data used in the text. This might include bam files, the reference fastqs used, and list of target sites. This would allow users to test if the software is working correctly. The github repository could also provide a more detailed description on the expected output.

 The authors might consider highlighting the limitations of their approach. For example, as it stands the method is most appropriate when only two taxa are possible. It is unclear how the approach would deal with a sample outside of the taxa pair e.g. if a horse bone was misidentified during morphological assignment as either sheep or goat. If the authors were interested, they could align a small number of the ancient horse samples to the sheep and goat genome and describe the observed results. The method may also struggle with samples showing cross contamination or when the genome pairs are closely related species (e.g. *Capra aegagrus* and *Capra nubiana* whose ranges overlap in the Early Holocene Levant). Again, this could be explored further if the authors were interested. 

**Comments**

**Method**

- Clarify in the text under “Target Sites” - the horse and donkey target sites are “type 1” only, correct?
- Clarify how “shared reads” between alignments are defined. Are they reads that share start and end positions between alignments? Are they reads which share id (first field in bam file)?
- Clarify the use of PMDtools in the pipeline - was it just used to check that the ancient samples were indeed ancient?
- In “Ancient DNA data processing”, there is some ambiguity as to how genotypes are called. For each target site, is the genotype of the *site* called or the “genotype” of the *read*? I personally would not describe reads as having “genotypes” - they carry bases representing an underlying genotype of the organism or genome. This also occurs under “Taxon assignment” - quoting the text “Each read can carry either reference or alternative genotypes at its target sites”. Alleles are also used to describe the base observed in a rad at a varying site, but genotype usually refers to the genome itself.
- Similarly under “Taxon assignment”, some clarity might be given that “the frequency of alternative alleles per read” refers to reads which overlap *more than one target site*. This is at least how I interpreted the sentence.

**Results**

- In the “Application to samples of known species identity”, there is ambiguity in the use of “accuracy”. Accuracy appears to refer to sample species assignment across the 18 tested ancient samples (“MTaxi yielded 100% accuracy for all 18 samples using the default approach”). Accuracy is also used to refer to a subset of the sample (“yielded 100% accuracy for all samples except one”; “100% accuracy was achieved for only n=4 sheep and n=8 goat”). Accuracy should be reported for the entire sample set e.g. 17/18=94.4% and 12/18=66.6%. An alternative reading was that the accuracy was the percentage of reads assigned to the correct species e.g. for shared reads, only 12 samples showed 100% of their reads assigned to their true species. Please clarify in the manuscript.

**Discussion**

- The method is described as both “elaborate” and “simple”. I would suggest the authors choose one! “Effective” might be a better word choice than “elaborate”. 
- In the final paragraph, please clarify that “mitochondrial introgression between the pairs of species” is what is meant.

Is the rationale for developing the new software tool clearly explained?

Yes

Is the description of the software tool technically sound?

Partly

Are sufficient details of the code, methods and analysis (if applicable) provided to allow replication of the software development and its use by others?

Partly

Is sufficient information provided to allow interpretation of the expected output datasets and any results generated using the tool?

Partly

Reviewer Expertise:

I work in the palaeogenomics of domestic animals, particularly goat. I have experience working with samples of extremely low endogenous DNA and sequencing reads, and have worked on species assignment of such samples. I was also responsible for the goat genomic data included in analyses in the text.

I confirm that I have read this submission and believe that I have an appropriate level of expertise to confirm that it is of an acceptable scientific standard, however I have significant reservations, as outlined above.

# Response

**Published:** 2023-06-18

**Authors:** Gözde Atağ

**Affiliations:** Middle East Technical University, Turkey

As the manuscript stands, I would recommend several changes that can be made to the text to improve clarity to the readers regarding the method. The authors might consider a revision of their manuscript to take on some or all of these suggestions, but the method itself appears robust. A major point of clarity is the input files required; while bam and fastqs are self explanatory, the files defining the transversion variant sites are less so. Should each file (for species 1 and 2) have the same number of variant sites, with line n in file 1 matching the same variant site in line n of file2? This could be clarified in the Github repository ( https://github.com/goztag/MTaxi).

**We thank Dr. Daly for pointing this out, we provided a more detailed explanation about the input files containing transversion variant sites in the Github repository as follows :** **“transv_poly_sp1 and transv_poly_sp2 should have the same number of variant sites, each line corresponding to the same site in both files”.**

 One additional request is for the inclusion in the Github repository of example data e.g. a small subset of data used in the text. This might include bam files, the reference fastqs used, and list of target sites. This would allow users to test if the software is working correctly. The github repository could also provide a more detailed description on the expected output.

**We now added sample data which includes all necessary files and results to the Github repository. We also provided an example of expected output in README.**

 The authors might consider highlighting the limitations of their approach. For example, as it stands the method is most appropriate when only two taxa are possible. It is unclear how the approach would deal with a sample outside of the taxa pair e.g. if a horse bone was misidentified during morphological assignment as either sheep or goat. If the authors were interested, they could align a small number of the ancient horse samples to the sheep and goat genome and describe the observed results. The method may also struggle with samples showing cross contamination or when the genome pairs are closely related species (e.g. Capra aegagrus and Capra nubiana whose ranges overlap in the Early Holocene Levant). Again, this could be explored further if the authors were interested.

**We thank Dr. Daly for this suggestion, we aligned n=3 ancient horse samples to sheep and goat references and tested how MTaxi behaves, the samples did not show any significant affinity to either species (p>0.05). We also tested how MTaxi deals with the pair of Capra aegagrus and Capra nubiana, n=2 Capra aegagrus samples were identified correctly (p<0.05) . We now added a section titled “Performance under alternative scenarios”, under which the results are presented. (Tables S5,S6)** **Table R1.** **MT** **axi results on horse genome data of known species identity (default approach), where horse is not among the candidate species.**

 Horse samples were aligned to sheep and goat references, and the analysis was performed with transversion substitutions between sheep and goat.

**Sample ID** **Taxon** **mtDNA** **coverage** **Total** **assigned** **reads** **Goat** **reads** **Sheep** **reads** **p value** **Identified** **taxon** Cdy2 Horse 0.319088 22 14 8 **>0.05** **-** Vhr031 Horse 0.72599 4 2 2 **>0.05** **-** Vhr102 Horse 1.29874 29 17 12 **>0.05** **-**  

**Table R2.** **MT** **axi results on goat ( *Capra aegagrus*) genome data of known species identity (default approach), where the candidate species are two goat species ( *Capra aegagrus* and *Capra nubiana*)** *C. aegagrus* samples were aligned to *C. aegagrus* and *C. nubiana* references, and the analysis was performed with transversion substitutions between *C. aegagrus* and *C. nubiana*. 

**Sample ID** **Taxon** **mtDNA** **coverage** **Total** **assigned** **reads** ***C.nubiana*** **reads** ***C.aegagrus*** **reads** **p value** **Identified** **taxon** Direkli1 *Capra aegagrus* 6.80549 1645 331 1314 **<0.001** *Capra aegagrus* Direkli6 *Capra aegagrus* 10.2286 2263 445 1818 **<0.001** *Capra aegagrus*      

 Comments   Method   Clarify in the text under “Target Sites” - the horse and donkey target sites are “type 1” only, correct?  

**We now specify that only ‘type 1’ target sites were used for horse and donkey.**

 Clarify how “shared reads” between alignments are defined. Are they reads that share start and end positions between alignments? Are they reads which share id (first field in bam file)?  

**We thank Dr. Daly for this point; we now define ‘shared reads’ as ones that share the same IDs between the two BAM files, under the “Ancient DNA data processing” section of the manuscript.**

 Clarify the use of PMDtools in the pipeline - was it just used to check that the ancient samples were indeed ancient?

**We now state that PMDtools was just used to check the authenticity of the ancient sequences in the Methods section as follows:** **“In order to confirm the authenticity of the ancient DNA sequences from non-UDG-treated libraries (TEP03, TEP62, TEP83, ULU26, ULU31), we evaluated damage patterns characteristic of aDNA using PMDtools (41) with the “--deamination” parameter; all libraries had >20% C->T and G->A damage at 5’ and 3’ ends, respectively.”**

 In “Ancient DNA data processing”, there is some ambiguity as to how genotypes are called. For each target site, is the genotype of the site called or the “genotype” of the read? I personally would not describe reads as having “genotypes” - they carry bases representing an underlying genotype of the organism or genome. This also occurs under “Taxon assignment” - quoting the text “Each read can carry either reference or alternative genotypes at its target sites”. Alleles are also used to describe the base observed in a rad at a varying site, but genotype usually refers to the genome itself.

**We agree with Dr. Daly about this ambiguity, we limited the use of the term ‘genotype’ to sites only, and used the term allele for the bases observed in a read.**

 Similarly under “Taxon assignment”, some clarity might be given that “the frequency of alternative alleles per read” refers to reads which overlap more than one target site. This is at least how I interpreted the sentence.

**We rewrote that section to avoid confusion. The manuscript now reads as follows:** **“MTaxi uses this genotype data to assign reads to either taxon, species 1 (SP1) or species 2 (SP2), based on whether they carry the alternative allele or not. If an SP1 read was aligned to the SP1 genome, we expect no alternative alleles at target sites, and if aligned to the SP2 genome, we expect all alternative alleles. For the reads which overlap more than one target site, MTaxi retains reads carrying only alternative alleles or carrying only reference alleles, thus excluding reads with inconsistent alleles (i.e. alternative and reference alleles mixed) at target sites.”** 

 Results   In the “Application to samples of known species identity”, there is ambiguity in the use of “accuracy”. Accuracy appears to refer to sample species assignment across the 18 tested ancient samples (“MTaxi yielded 100% accuracy for all 18 samples using the default approach”). Accuracy is also used to refer to a subset of the sample (“yielded 100% accuracy for all samples except one”; “100% accuracy was achieved for only n=4 sheep and n=8 goat”). Accuracy should be reported for the entire sample set e.g. 17/18=94.4% and 12/18=66.6%. An alternative reading was that the accuracy was the percentage of reads assigned to the correct species e.g. for shared reads, only 12 samples showed 100% of their reads assigned to their true species. Please clarify in the manuscript.

**We thank Dr. Daly for pointing out this ambiguity. We now report accuracy for the entire sample set. We also added a section called “Performance metrics” to Methods, in which the calculation of accuracy is explained.** 

 Discussion   The method is described as both “elaborate” and “simple”. I would suggest the authors choose one! “Effective” might be a better word choice than “elaborate”.

**We thank Dr. Daly for this suggestion, we now describe the method as simple and effective.** 

 n the final paragraph, please clarify that “mitochondrial introgression between the pairs of species” is what is meant.

**We clarified that we mean mitochondrial introgression between the pairs of species.**         
